# Nucleoside Analogues as Antibacterial Agents

**DOI:** 10.3389/fmicb.2019.00952

**Published:** 2019-05-22

**Authors:** Jessica M. Thomson, Iain L. Lamont

**Affiliations:** Department of Biochemistry, University of Otago, Dunedin, New Zealand

**Keywords:** repurposed antibiotics, antibiotic resistance, pyrimidine analogues, purine analogues, multidrug-resistant bacteria, antibacterial agents, antimicrobial

## Abstract

The rapid increase in antibiotic-resistant bacteria has emphasized the urgent need to identify new treatments for bacterial infections. One attractive approach, reducing the need for expensive and time-consuming clinical trials, is to repurpose existing clinically approved compounds for use as antibacterial agents. Nucleoside analogues are commonly used for treating viral and fungal infections, as well as for treating cancers, but have received relatively little attention as treatments for bacterial infections. However, a significant number of clinically approved derivatives of both pyrimidines and purines including halogenated, thiolated, and azolated compounds have been shown to have antibacterial activity. In the small number of studies carried out to date, such compounds have shown promise in treating bacterial infections. Here, we review the mechanisms of action and antibacterial activities of nucleoside analogues that can potentially be repurposed for treating infections as well as considering possible limitations in their usage.

## Introduction

The introduction of antibiotics into clinical use heralded a new age for medicine. However, less than a century later, the therapeutic efficacy of antibiotics is becoming limited owing to the rise of resistance in pathogenic bacteria (Lewis, 2013). Once a problem largely limited to hospital environments, antibiotic-resistant strains of pathogens have progressively become more prevalent in the community, and their spread has been unrestricted (Rice, 2009). It has been estimated that there are at least 700,000 deaths a year worldwide due to infections by antibiotic-resistant bacteria and in the absence of new treatment strategies this figure could rise to 10 million deaths a year by 2050 (O’Neill, 2014). Despite an urgent need for new antibiotics to combat resistant pathogens, there have been very few novel antibiotics to make it into clinical practice. Indeed, the majority of antibiotics in use today belong to classes discovered before the 1970s (Lewis, 2013). There are a number of factors contributing to the issue of failing antibiotic discovery, including declining interest in discovery by pharmaceutical companies due to the high costs of bringing drugs to market and limited approaches to identify lead compounds (Livermore, 2011). However, some promising approaches to antibiotic discovery do exist. Recently, novel methods of screening environmental microbes have yielded promising antibiotic compounds (Gavrish et al., 2014; Ling et al., 2015). While such screening techniques hold promise for discovery of novel compounds, it will be a number of years before any discovered compounds are sufficiently studied to be used clinically. Thus, a different approach to discovery, namely drug repurposing, may identify compounds with antibiotic activity and represents a way to fast track them into clinical use (Brown, 2015; Rangel-Vega et al., 2015; Miro-Canturri et al., 2019). The concept behind repurposing is that almost all drugs in clinical use exhibit various pharmacological activities secondary to their main activity. Screening these compounds for side activities, and optimizing activity if required, could represent a way to identify new antibiotics. As the safety profiles of the drugs are known, clinical trials could be less extensive, thus reducing costs associated with approval and allowing drugs to be used clinically more rapidly than completely novel drugs.

One class of drugs that are important from a clinical perspective is nucleoside analogues, a pharmacologically diverse class of drugs that arose from chemically modified natural ribose or 2′-deoxyribose nucleosides (Koszytkowska-Stawinska and Buchowicz, 2014). Nucleoside analogues are among the most important drugs in the clinical setting and are used widely as both anticancer and antiviral agents (Jordheim et al., 2013). Nucleoside analogues mimic endogenous nucleosides, exploiting cellular metabolism and becoming incorporated into both DNA and RNA. This property makes nucleoside analogues effective at inhibiting viral replication and stopping cancer cell proliferation. However, although there have been some studies on the efficacy of nucleoside analogues as antibacterial agents, the potential use of these compounds in treating bacterial infections has been relatively neglected.

For the purposes of this review, a nucleoside analogue is defined as a nucleobase linked to a sugar, where the nucleobase or the sugar component is altered such that the molecule becomes distinct from those found naturally. Modifications to nucleobases include halogenation and addition of azido groups, and modifications of the sugar component include ring opening, halogenation, methylation, and hydroxylation or dehydroxylation (Jordheim et al., 2013). It is worth noting that a class of drugs called nucleoside antibiotics has also been studied for potential clinical use as antibacterials. Nucleoside antibiotics typically consist of parts of natural nucleosides conjugated with additional complex structures such as amino acids or fatty acids, with the combination having antibacterial properties. In bacteria, nucleoside antibiotics primarily target cell-wall biosynthesis (Winn et al., 2010; Niu and Tan, 2015). The potential use of various nucleoside antibiotics has been extensively reviewed elsewhere (Winn et al., 2010; Carter and McDonald, 2014; Niu and Tan, 2015) and is beyond the scope of this review.

**Figure 1:**
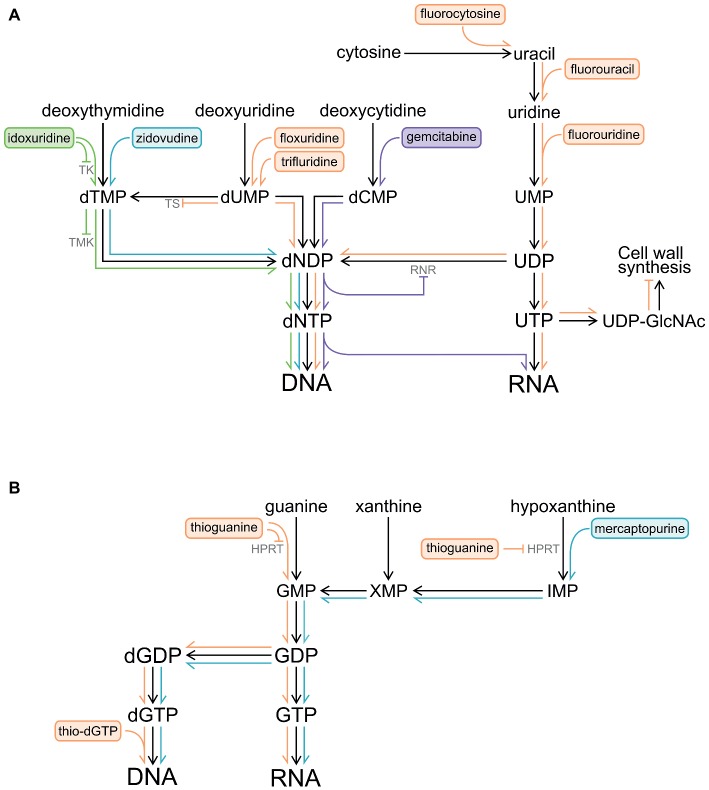
Pathways by which pyrimidine and purine analogues are metabolized and become incorporated into DNA and RNA. Inhibition of enzymes by nucleoside analogues and their metabolites is also shown (⊣). **(A)** Pyrimidine analogues. **(B)** Purine analogues. Abbreviations: HPRT, hypoxanthine-guanine phosphoribosyl transferase; IMP, inosine monophosphate; RNR, ribonucleotide reductase; TK, thymidylate kinase; TMK, deoxythymidine monophosphate kinase; TS, thymidylate synthase; UDP-GlcNAc, uridine diphosphate N-acetylglucosamine; XMP, xanthosine monophosphate.

This review will cover nucleoside analogues that have shown clinical potential for repurposing as antibacterials. A summary of the metabolism of the relevant compounds is shown in Figure 1. First, their usage and modes of action in treating cancer or viral infections, along with studies validating their antibiotic activity against bacteria, will be outlined. Second, studies on the metabolism of nucleoside analogues and their mechanism of action in bacteria will be discussed. Finally, limitations of nucleoside analogues as antibiotics including potential for toxicity and development of resistance will be considered.

## Pyrimidine Analogues

### Gemcitabine

**Figure 2:**
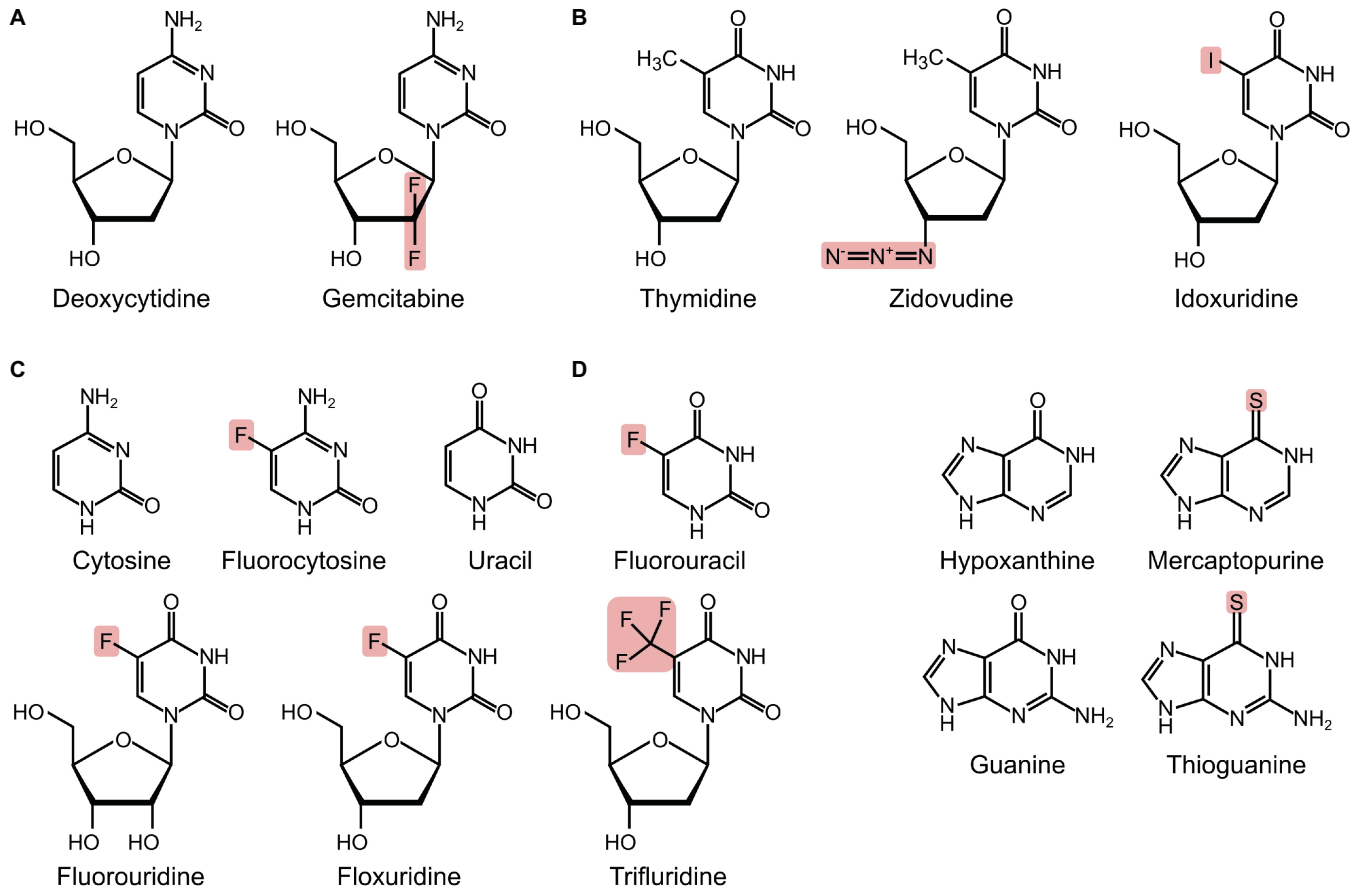
Structures of pyrimidines, purines, and their analogues, with substituted atoms highlighted. **(A)** Deoxycytidine and its analogue. **(B)** Thymidine and its analogues. **(C)** Cytosine, uracil, and fluorinated pyrimidines. **(D)** Hypoxanthine, guanine, and their analogues.

Gemcitabine (2′,2′-difluoro-2′-deoxycytidine) is an analogue of deoxycytidine and has two fluorine atoms in place of hydrogen atoms on the 2′ carbon of the sugar component (Figure 2A). Gemcitabine was first developed as an antiviral drug but has since been used exclusively as an anticancer drug (Mini et al., 2006). Gemcitabine is used alone or in combination with other drugs for various types of cancers, including metastatic pancreatic cancer (Burris et al., 1997; Ishii et al., 2005). Gemcitabine is a prodrug, which is taken up into eukaryotic cells, phosphorylated to gemcitabine monophosphate by deoxycytidine kinase, and converted to the active metabolites gemcitabine di- and triphosphate (Heinemann et al., 1988; Mini et al., 2006). Once activated, gemcitabine is incorporated into growing DNA (Figure 1), ultimately resulting in termination of strand elongation. After incorporation of gemcitabine, the DNA polymerase adds one additional deoxynucleotide and DNA synthesis then ceases; the additional deoxynucleotide may mask gemcitabine from proof-reading exonucleases, preventing its removal (Huang and Plunkett, 1995). It is unclear why only one nucleotide is added after gemcitabine, but it may be that the drug induces DNA conformation changes, preventing the addition of further nucleotides (Plunkett et al., 1995). Gemcitabine can also potently inhibit ribonucleotide reductase (Figure 1; Huang and Plunkett, 1995). There is evidence that a metabolite of gemcitabine can be incorporated into RNA, although the identity of this metabolite and the impact this has on cells are unclear (Figure 1; Vanhaperen et al., 1993).

Gemcitabine has been the subject of various repurposing studies. In a study that explored the efficacy of various nucleoside analogues against clinical isolates from different bacteria genera, gemcitabine was identified as having antibacterial activity (Sandrini et al., 2007b). Gemcitabine had a potent antibacterial effect on Gram-positive bacteria, including important pathogenic species from genera *Listeria*, *Bacillus*, *Enterococcus,* and *Staphylococcus,* although it was ineffective against Gram-negative bacteria. A murine infection model was used to investigate whether gemcitabine was useful as an antibiotic *in vivo* (Sandrini et al., 2007a). Mice were infected with *Streptococcus pyogenes* AP1, a virulent strain responsible for causing the majority of severe *S. pyogenes* infections. Of the mice infected with a potentially fatal dose of *S. pyogenes* AP1, those treated with the control had a 100% mortality rate, whereas those treated with gemcitabine had only a 17% mortality rate. This demonstrated that gemcitabine had potent activity against *S. pyogenes in vivo*.

These studies showed gemcitabine to be a good candidate for repurposing as an antibiotic. Jordheim et al. (2012) performed additional *in vitro* preclinical studies to investigate the potential of gemcitabine, especially its efficacy against clinically important multidrug-resistant strains of *Staphylococcus aureus*. Gemcitabine was found to be active against 19 different strains of methicillin-resistant *S. aureus*. Gemcitabine was effective against glycopeptide-intermediate *S. aureus*, a strain resistant to all glycopeptide antibiotics, including vancomycin. Resistance to gemcitabine could develop in treated *S. aureus* (Jordheim et al., 2012). However, gemcitabine was found to have synergistic activity with gentamicin, and if used in combination, emergence of resistance to these drugs may be slowed (Jordheim et al., 2012).

### Zidovudine

Zidovudine [3′azido-3′-deoxythymidine, AZT] is a thymidine analogue with an azido group in place of the hydroxyl group at the 3′ carbon of the deoxyribose ring (Figure 2B). Zidovudine is an antiretroviral agent and is used clinically as therapy for HIV/AIDS. Once activated to its triphosphorylated form, zidovudine inhibits viral replication. The azido group of zidovudine prevents phosphodiester bond formation and results in DNA chain termination (Furman et al., 1986; Cooper and Lovett, 2011). Zidovudine is effective as an antiretroviral because it has an affinity for the viral reverse transcriptase that is approximately 100-fold greater than its affinity for human DNA polymerase (Furman et al., 1986). However, zidovudine has still been found to be incorporated into the DNA of patients taking the drug, and there is evidence that at high doses, zidovudine can lead to various toxicities, including mitochondrial toxicity and cardiomyopathy (Lewis et al., 1992, 2000).

Zidovudine was first found to have antibacterial activity in the late 1980s. Zidovudine had antimicrobial activity against various Enterobacteriaceae, including *Salmonella* species (Elwell et al., 1987). Zidovudine was activated in these bacteria by thymidine kinase (TK), and incorporation of activated zidovudine into bacterial DNA resulted in DNA chain termination (Figure 1; Elwell et al., 1987). Subsequently, zidovudine has been demonstrated to have potent activity against many pathogenic Gram-negative bacteria, including *Escherichia coli*, *Salmonella typhimurium*, *Klebsiella pneumoniae*, *Shigella flexneri,* and *Haemophilus influenzae* and including isolates resistant to conventional antibiotics (Keith et al., 1989; Sandrini et al., 2007a,b; Doleans-Jordheim et al., 2011; Peyclit et al., 2018). It also acts synergistically with conventional antibiotics, enhancing their effectiveness (Wambaugh et al., 2017; Ng et al., 2018; Falagas et al., 2019; Hu et al., 2019). Zidovudine is ineffective against Gram-positive bacteria such as *Listeria* species, *Bacillus* species, Staphylococci, and *Enterococcus faecalis* as well as against *Mycobacteria* species and *Pseudomonas aeruginosa* (Elwell et al., 1987; Sandrini et al., 2007a).

The antibacterial activity of zidovudine has been demonstrated both *in vitro* and *in vivo*. Herrmann and Lagrange (1992) used a macrophage cell line to demonstrate that zidovudine inhibited intracellular growth of *S. typhimurium*. Zidovudine had potent *in vivo* activity. Zidovudine prevented lethal infections in mice with pyelonephritis caused by *E. coli* infection, being as effective as either trimethoprim or ampicillin (Keith et al., 1989). It also inhibited growth of antibiotic-resistant *E. coli* and *K. pneumoniae* in a murine peritoneal infection model, acting synergistically with colistin (Hu et al., 2019). When administered subcutaneously, zidovudine also prevented lethal salmonellosis in calves infected with *S. dublin* (Keith et al., 1989). Zidovudine has therapeutic potential for humans as well; zidovudine given as an antiretroviral to HIV/AIDS patients also had the additional protective effect of lowering the recurrence of *Salmonella* bacteremia, a significant problem for HIV/AIDS patients (Casado et al., 1999). These *in vivo* findings suggest that zidovudine has potential application as an antibacterial agent. Zidovudine has also been the subject of modification studies, which aim to improve its therapeutic efficacy and resolve issues like short half-life of the drug. Research has gone into creating zidovudine derivatives that retain antiviral activity while having improved bactericidal activity (Moroni et al., 2002). Such derivatives may be particularly useful for HIV/AIDS patients; HIV/AIDS patients are susceptible to opportunistic bacterial infections, and improved bactericidal profile of these derivatives would be a beneficial side activity.

### Fluorinated Pyrimidines

Originally synthesized as antitumor drugs (Heidelberger et al., 1957), fluorinated pyrimidines have also been used widely as antifungals (Vermes et al., 2003), have some use as antivirals (Wilhelmus, 2010), and show promise as antibacterials. The fluorinated pyrimidine family was first synthesized after the observation that tumor cells preferentially utilized uracil for nucleic acid biosynthesis (Rutman et al., 1954; Heidelberger et al., 1957). From this large family of compounds, the nucleobase 5-fluorouracil and the nucleoside floxuridine (5-fluoro-2′deoxyuridine, Figure 2C) are frequently used for the treatment of various cancers (Galmarini et al., 2002; Alvarez et al., 2012). While these compounds are effective anticancer drugs and are taken up more rapidly by cancerous cells, they also affect non-cancerous cells, and their use is associated with a number of side effects, such as bone marrow depression (Galmarini et al., 2002). Trifluridine (Figure 2C) is used as an antiviral and is a therapy for herpetic simplex virus epithelial keratitis (Wilhelmus, 2010). 5-Fluorocytosine (Figure 2C), a prodrug of 5-fluorouracil, is used as an antifungal agent. 5-Fluorocytosine is most commonly used in combination therapy with other antifungals, typically amphotericin B, as resistance to 5-fluorocytosine arises readily (Bennett, 1977; Francis and Walsh, 1992; Ghannoum and Rice, 1999). 5-Fluorocytosine itself is not active; antifungal activity is dependent upon uptake of 5-fluorocytosine into fungal cells and subsequent deamination to 5-fluorouracil (Figure 1; Bennett, 1977). In turn, 5-fluorouracil is converted to floxuridine mono-, di-, and tri-phosphate. Metabolites of 5-fluorouracil and floxuridine can become incorporated into DNA and RNA and can also inhibit thymidylate synthase, preventing *de novo* formation of deoxythymidine monophosphate (Figure 1; Bennett, 1977).

Compounds from the fluorinated pyrimidine family have antibacterial activity. Various fluorinated pyrimidines could inhibit the growth of the human pathogen *Ureaplasma urealyticum* (Carnrot et al., 2003). Floxuridine had a strong effect *in vitro*, inhibiting the growth of *U. urealyticum* for up to 72 h. This finding was supported by a later study (Wehelie et al., 2004). Subsequently, various Gram-positive and Gram-negative bacteria were found to be susceptible to floxuridine (Sandrini et al., 2007a,b; Zander et al., 2010a). Gram-positive bacteria, such as various streptococci, *S. aureus* and *Bacillus* species, were more sensitive than the Gram-negative bacteria tested. Floxuridine has also been found to have synergistic effects when used in combination with zidovudine (Wambaugh et al., 2017). 5-Fluorouracil has been reported to decrease virulence of *P. aeruginosa* by disrupting biofilm formation (Ueda et al., 2009) and also has activity against *Mycobacterium tuberculosis* and a number of oral microbes including the pathogen *S. pyogenes* (Singh et al., 2015; Vanlancker et al., 2016). Floxuridine and trifluridine both affected the growth of *Mycoplasma pneumoniae*, with trifluridine strongly inhibiting growth *in vitro* (Sun and Wang, 2013). These studies highlight the antibacterial activity of fluorinated pyrimidines.

The possible application of fluorinated pyrimidines as antibacterials has been tested *in vivo* (Walz et al., 2010; Imperi et al., 2013; Kirienko et al., 2016). 5-Fluorouracil was demonstrated to be a clinically useful antibacterial agent by Walz et al. (2010). In a phase III clinical trial, Walz and colleagues studied the efficacy of central venous catheters coated with 5-fluorouracil in decreasing catheter-related bloodstream infections and reducing colonization of catheters by bacteria. Nearly 1,000 patients were randomized into two groups, one receiving a catheter coated with 5-fluorouracil and the other group receiving standard catheters coated with chlorhexidine-silver sulfadiazine (Walz et al., 2010). The catheters coated with 5-fluorouracil were as effective at preventing bacterial colonisation as the standard chlorhexidine-silver sulfadiazine catheters. The frequency of adverse events was comparable between the two groups. The dose of 5-fluorouracil used to coat the catheter was very low, and in a small tissue sample obtained from the location of catheterization, no toxicity was observed in patients receiving 5-fluorouracil-coated catheters (Walz et al., 2010). These data suggest that if widespread resistance to compounds currently impregnated in standard catheters were to arise, then 5-fluorouracil impregnated catheters could be clinically useful as an alternative. It is also encouraging that the 5-fluorouracil coating the catheters did not cause any toxicity or significant side effects.

While 5-fluorouracil has been shown to be a potentially effective antimicrobial, the drug does not specifically target bacteria and is also toxic to eukaryotic cells (Alvarez et al., 2012). Conversely, 5-fluorocytosine is a prodrug and is only converted to 5-fluorouracil by susceptible cells. Human cells lack the enzymes required to convert 5-fluorocytosine to 5-fluorouracil; therefore, the drug is less toxic than 5-fluorouracil to patients. The potential of 5-fluorocytosine as an antibacterial was identified from a drug repurposing screen. 5-Fluorocytosine was identified as a compound that inhibited production of a number of virulence factors of *P. aeruginosa* (Imperi et al., 2013, 2019). 5-Fluorocytosine suppressed *P. aeruginosa* pathogenicity in a mouse model of pulmonary infection (Imperi et al., 2013) as well as in a *Caenorhabditis elegans* infection model (Kirienko et al., 2016). 5-Fluorocytosine needs to be deaminated to 5-fluorouracil to inhibit virulence factor production of *P. aeruginosa* (Imperi et al., 2013, 2019).

### Idoxuridine

Idoxuridine (5-iodo-2′-deoxyuridine; Figure 2B) was the first antiviral drug introduced to the clinic (Kaufman, 1962); nowadays, it is mostly used in the therapy of herpetic simplex keratitis (Wilhelmus, 2010). At high concentrations, idoxuridine is cytotoxic because it competitively inhibits various enzymes, including TK, and when triphosphorylated can become incorporated into DNA (Figure 1; Prusoff et al., 1979). The exact effect of the incorporation of triphosphorylated idoxuridine into DNA is unknown.

Idoxuridine alone had no antimicrobial properties against a range of clinically important pathogens tested (Zander et al., 2010a). However, when used in conjunction with SXT, a combination of trimethoprim and sulfamethoxazole that inhibits tetrahydrofolic acid synthesis, idoxuridine significantly enhanced the antimicrobial properties of SXT against most of the bacterial species tested, including *K. pneumoniae*, *S. aureus,* and *S. pyogenes* (Zander et al., 2010a,b). Tetrahydrofolic acid is a critical bacterial cofactor of thymidine synthesis, and thus, DNA synthesis (Sköld, 2009) and inhibition of TK by idoxuridine evidently enhanced the effects of SXT in inhibiting DNA synthesis. This was the case even in the presence of extracellular thymidine, which would be present during infections and ameliorate the effects of SXT (Zander et al., 2010a,b).

While the combination of idoxuridine and SXT has promise for clinical application, extensive clinical studies will need to be performed. The use of idoxuridine is severely limited because of its cytotoxicity, and its use is largely limited to topical applications, where toxicity is not significant (Boston Interhospital Virus Study Group and NIAID-Sponsored Cooperative Antiviral Clinical Study, 1975; Yolton and Haesart, 2008). Although idoxuridine was effective against *S. aureus in vitro* at concentrations below those known to be cytotoxic (Zander et al., 2010a), it is unclear whether such low concentrations would improve the bactericidal effect of drugs like SXT *in vivo*.

## Purine Analogues

### Thiopurines

In contrast to pyrimidines, for which halogenated analogues have been characterized, purine analogues with potential application as antimicrobials are predominantly thio-derivatives. Currently, the predominant clinical uses for thiopurines are as anticancer, immunosuppressant, and anti-inflammatory agents. In particular, they are important therapeutic agents for acute leukemias and for inflammatory bowel disease (Galmarini et al., 2002; Cooper and Brown, 2015; Hanauer et al., 2019). Compounds from the thiopurine family (Figure 2D) have antibacterial activity. As mercaptopurine has *in vitro* activity against *Mycobacterium avium* subspecies *paratuberculosis* and *Corynebacterium* species (Greenstein et al., 2007; Shin and Collins, 2008; Liu et al., 2017), 6-thioguanosine 5′-triphosphate (thio-dGTP) can inhibit spore germination of *Bacillus anthracis* in an infected macrophage cell line (Akoachere et al., 2007; Alvarez et al., 2010), and thioguanine can inhibit *in vitro* growth of *Mycoplasma pneumoniae* (Sun and Wang, 2013).

Mercaptopurine is an analogue of hypoxanthine, and thioguanine is an analogue of guanine. Mercaptopurine and thioguanine require phosphorylation before they can exert their therapeutic effects. Both mercaptopurine and thioguanosine are metabolized intracellularly to the active thio-dGTP (Lennard, 1992), although mercaptopurine is also metabolized to other nucleoside derivatives. Thio-dGTP exerts its cytotoxic effect on cells primarily by becoming incorporated into DNA (Figure 1), resulting in local changes to the DNA structure, largely because it forms an unstable base pair with deoxycytidine (Somerville et al., 2003; de Boer et al., 2007; Karran and Attard, 2008). This change in structure can lead to DNA breaks and also inhibition of DNA replication (Somerville et al., 2003). Thiopurine derivatives can also inhibit enzymes involved in *de novo* purine synthesis (Figure 1; Galmarini et al., 2002). In eukaryotic cells, thio-dGTP is also known to interfere with secondary messengers and energy carrying processes, competing with natural guanosine triphosphate (de Boer et al., 2007).

*M. avium* ssp. *paratuberculosis* may be an environmental trigger for Crohn’s disease or even a causative agent of the disease (Greenstein and Collins, 2004). Mercaptopurine is commonly used to induce and maintain remission of Crohn’s disease. The effect of mercaptopurine on the growth of *M. avium* ssp. *paratuberculosis* was therefore tested, and the drug was found to inhibit growth *in vitro* (Greenstein et al., 2007; Shin and Collins, 2008), although *in vivo* experiments have yet to be carried out.

Inosine is an important germinant of *B. anthracis in vitro*. Inosine analogues and guanosine analogues were tested for their ability to block *in vitro* germination of spores (Akoachere et al., 2007). Thio-dGTP was the only compound identified as being effective at preventing spore germination. The ability of thio-dGTP to prevent necrosis in murine macrophages infected with *B. anthracis* was subsequently tested (Alvarez et al., 2010). Thio-dGTP and its parent compound 6-thioguanine were the most effective analogues and were able to protect cells from necrosis even after the time point at which 100% cell death normally occurs (Alvarez et al., 2010). While the activity of thio-dGTP as an antigerminant is promising, spore germination *in vivo* is a complex process dependent upon numerous factors, and further studies of the efficacy of thio-dGTP are required.

## Metabolism and Mechanisms of Action of Nucleoside Analogues in Bacteria

Pyrimidine and purine analogues utilize the same pathways as their natural counterparts and therefore compete for both uptake and metabolism (Sun and Wang, 2013). Typically, nucleobase and nucleoside analogues enter bacterial cells through membrane transporters and then are cycled through the nucleotide salvage pathway, where they are activated by deoxyribonucleoside kinases (dNKs). dNKs perform the first committed reaction in the salvage pathway, namely the phosphorylation of deoxyribonucleosides (Figure 1). In bacteria, the metabolism and mechanism of action of pyrimidine and purine analogues have significant differences and will be further discussed separately.

### Pyrimidine Analogues

Early work on the metabolism and mechanism of action of pyrimidine analogues in bacteria demonstrated the importance of the enzymes that metabolize them, such as dNKs. Thymidine kinase (TK), a dNK, was shown to be necessary for activation of zidovudine in bacteria and subsequent incorporation of activated zidovudine into the DNA (Elwell et al., 1987). Since the identification of TK as being important for the activity of zidovudine, the involvement of dNKs and other enzymes on the metabolism and activity of pyrimidine analogues has been demonstrated.

dNK genes are differentially distributed among different bacterial species (Saito and Tomioka, 1984; Sandrini et al., 2007b; Konrad et al., 2012), and this differential distribution explains in part species-specific susceptibilities to nucleoside analogues. For example, *P. aeruginosa* lacks TK activity and is not susceptible to many pyrimidine analogues that have activity against other Gram-negative bacteria. Additionally, the presence of different dNKs in different species explains differences in responses to gemcitabine, which is found to be effective predominantly against Gram-positive bacteria. *E. coli* that lacks deoxyadenosine kinase is not usually susceptible to gemcitabine but is sensitive to this compound when expressing deoxyadenosine kinases from Gram-positive bacteria such as *S. aureus*, *S. pyogenes*, and *B. cereus* (Sandrini et al., 2007a,b).

Zidovudine is effective against some Gram-negative but not Gram-positive species (Sandrini et al., 2007a). The basis for this was explored using an *E. coli* mutant lacking TK. This mutant was resistant to zidovudine but became sensitized when transformed with TK genes from either Gram-negative species or zidovudine-resistant Gram-positive species such as *Bacillus cereus* and *Listeria monocytogenes* (Elwell et al., 1987; Sandrini et al., 2007a,b). These findings suggest that resistance in the Gram-positive species is not a result of lack of TK activity toward zidovudine, but instead that the monophosphate form of zidovudine resulting from the action of TK is a poor substrate for subsequent enzymes such as thymidylate kinase (Sandrini et al., 2007a). TKs are also important for the activity of other fluorinated pyrimidines such as floxuridine in *U. urealyticum* (Carnrot et al., 2003). Floxuridine and trifluridine are substrates for *U. urealyticum* TK and are converted to the monophosphate forms that may be further metabolized and incorporated into DNA (Sun and Wang, 2013). The monophosphate forms of floxuridine and trifluridine may also inhibit thymidylate synthase.

An important part of the mechanism of action of nucleoside analogues in cancers and viruses is the incorporation of their metabolites into nucleic acids. There is evidence that this incorporation is important in bacteria as well. Metabolites of 5-fluorouracil become incorporated into *E. coli* RNA and to a lesser extent DNA (Warner and Rockstroh, 1980). The proportion of fluorinated uracil derivatives in the DNA increased significantly when the enzymes responsible for the removal of deoxyuridine triphosphate (UTP) from DNA were absent, indicating that this mechanism has a role in the removal of fluorinated UTP from DNA (Warner and Rockstroh, 1980). Fluorinated metabolites were also detected in the nucleic acids of *Mycobacterium tuberculosis* (Singh et al., 2015). In *M. tuberculosis*, treatment with 5-fluorouracil also led to an upregulation of DNA damage response genes proposed to be a result of DNA breakages caused by incorporation of fluorinated UTP (Singh et al., 2015).

An effect of fluorinated pyrimidines independent of nucleic acid incorporation has also been observed. Treatment with 5-fluorouracil caused *E. coli* to become osmotically sensitive, leading to a rapid loss of bacterial viability (Tomasz and Borek, 1959), an effect proposed to be due to metabolites of 5-fluorouracil affecting UDP-linked cell-wall precursors (Tomasz and Borek, 1960, 1962). 5-Fluorouracil partially inhibited peptidoglycan biosynthesis in *S. aureus,* leading to accumulation of fluorinated cell-wall precursors (Rogers and Perkins, 1960; Stickgold and Neuhaus, 1967). Accumulation of fluorinated cell-wall precursors also occurred in 5-fluorouracil-treated *Mycobacterium tuberculosis* (Singh et al., 2015).

### Purine Analogues

One of the first enzymes in the purine salvage pathway is hypoxanthine guanine phosphoribosyl transferase (HPRT) (Figure 1). Thioguanosine strongly inhibited the growth of *M. pneumoniae*, and HPRT is a target of this analogue (Sun and Wang, 2013). Thioguanosine strongly inhibited uptake of hypoxanthine and guanosine and their subsequent incorporation into DNA and RNA. Thioguanosine also strongly inhibited activity of *M. pneumoniae* HPRT with either of its natural substrates, hypoxanthine and guanosine (Figure 1). Thioguanosine inhibited *M. pneumoniae* HPRT much more effectively than human HPRT. This likely reflects structural differences between humans and *M. pneumoniae* HPRT leading to differences in binding of thioguanosine; these differences may allow for designing pathogen-specific HPRT inhibitors (Sun and Wang, 2013).

## Limitations of Nucleoside Analogues as Antibiotics

### Toxicity

While the safety profiles of clinically used nucleoside analogues are generally favorable, some of these drugs do have side effects. Nucleoside analogues, especially those used as anticancer agents, are often not specific for their target cells and also affect healthy human cells. Therefore, the use of this class of drugs is associated with a number of side effects. Many side effects are mild and do not require cessation of treatment, but some side effects are severe. For example, some analogues such as trifluridine and idoxuridine are only used topically as they have severe side effects when used systemically. While severe side effects as a result of treatment with nucleoside analogues are generally rare, they will need to be considered if nucleoside analogues are to have clinical application as antibiotics.

One of the most common significant side effects of nucleoside analogues is myelosuppression. Myelosuppression is a decrease in the production of blood cells in bone marrow and can affect all types of blood cells. A serious type of myelosuppression caused by chemotherapy is neutropenia, which is the reduction of leukocytes. Neutropenia predisposes patients to infections and is the dose-limiting factor in many therapeutic regimens with nucleoside analogues, including gemcitabine, 5-fluorouracil, and floxuridine, and the thiopurines (Galmarini et al., 2002). Myelosuppression is also a dose-limiting toxicity in the use of 5-fluorocytosine as an antifungal agent. Even though 5-fluorocytosine is a prodrug and human cells lack the enzyme needed to convert it to 5-fluorouracil, when serum concentration of 5-fluorocytosine reaches 100 μg/ml or higher, toxicities similar to those seen in patients taking 5-fluorouracil can occur (Kauffman and Frame, 1977; Harris et al., 1986). Why these toxicities occur is unclear, although there is evidence that the host gut microbiota may convert 5-fluorocytosine to 5-fluorouracil, which can cause myelosuppression (Harris et al., 1986; Vermes et al., 2003). Of the drugs discussed in this review, idoxuridine highlights the problem myelosuppression may cause when nucleoside analogues are used as antibiotics. While idoxuridine is used to treat herpetic simplex keratitis of the eye, it has been found to worsen and slow healing of bacterial infections of the eye (Yamaguchi et al., 1979). Thus, nucleoside analogues that need to be administered at doses that cause myelosuppression may be contraindicated for use against bacterial infections.

Another side effect is pulmonary toxicity, commonly associated with gemcitabine use. Gemcitabine causes a range of pulmonary toxicities, from those that are mild and do not require stopping treatment, to rare but severe side effects, like pulmonary fibrosis and respiratory distress syndrome (Chi et al., 2012). It is unclear why gemcitabine has this effect, but it has been proposed that pulmonary toxicity occurs more frequently in patients who have underlying pulmonary disease or in patients who are concomitantly taking therapeutics that can also cause pulmonary toxicity (Gupta et al., 2002). The concentrations of gemcitabine that inhibited the growth of *S. aureus in vitro* were lower than the plasma concentrations of gemcitabine in cancer patients, suggesting that lower doses than are used in oncology may be effective in treating infections, potentially reducing side effects of gemcitabine (Jordheim et al., 2012).

While it is proposed that nucleoside analogues would be effective against bacteria at concentrations that make them unlikely to cause myelosuppression or other serious side effects (Walz et al., 2010; Zander et al., 2010a; Jordheim et al., 2012), toxicity remains a serious consideration that must be resolved before these drugs can be used as antibacterials. Treatment of bacterial infections with compounds, which at high serum concentrations can have immunomodulatory or toxic effects, could potentially result in the drug exacerbating the infection it was intended to treat. The examples described above highlight the fact that a significant amount of investigation into the safety of nucleoside analogues as antibiotics will have to be performed to ensure bacterial infections are not worsened by administration of analogues.

A potential way to overcome any toxic effects is to search for nucleoside analogues that are more specific for their bacterial targets. Characterization of the substrate specificities of different bacterial dNKs, which are responsible for the activation of many nucleoside analogues in bacteria, suggests that it may be possible to design analogues specific for bacterial dNKs (Sandrini et al., 2007a; Doleans-Jordheim et al., 2011).

### Resistance

As found for classical antibiotics, resistance can arise when using nucleoside analogues as antibiotics. Resistance to zidovudine and gemcitabine has been studied in detail. After short-term exposure to zidovudine, *Salmonella* and *E. coli* strains have been found develop stable high-level resistance both *in vitro* and *in vivo* (Lewin et al., 1990a,b; Doleans-Jordheim et al., 2011). Zidovudine-resistant *E. coli* has been isolated from HIV/AIDS patients taking zidovudine as antiretroviral therapy, whereas *E. coli* isolated from control samples was susceptible (Lewin et al., 1990b, 1991).

A mechanism for resistance to zidovudine was first proposed by Lewin et al. (1990a). It was found that zidovudine-resistant strains of *E. coli* and *S. typhimurium* were unable to incorporate radiolabeled thymidine into their chromosomal DNA, and that this incorporation was still prevented in the presence of a membrane permeabilizer. This suggested that in these bacteria, resistance was due not to inhibition of uptake but rather to loss of TK activity (Lewin et al., 1990a). As TKs have been shown to be important for conversion of nucleoside analogues into their active forms, this may provide an explanation for the observed resistance. Zidovudine has been found to induce mutations in *E. coli* (Doleans-Jordheim et al., 2011). Because of the apparent importance of TK on the metabolism of zidovudine (Sandrini et al., 2007a,b), Doleans-Jordheim et al. (2011) compared the sequence of the TK-encoding gene from resistant strains and susceptible strains. All 10 strains analyzed had changes to the sequences of the gene that would result in changes to the TK protein, including amino acid changes and premature stop codons.

In addition to zidovudine resistance *via* a non-functional TK enzyme, there appears to be another mechanism of resistance. Some resistant bacteria, like staphylococci, do have functional TK enzymes (Lewin et al., 1990a). The reason why these bacterial strains are zidovudine resistant is not clear, but it is possible that in such strains, zidovudine does not act as a substrate for TK or DNA polymerase and is thus not incorporated into nucleic acids (Lewin et al., 1990a).

Resistance to gemcitabine has also been observed. Jordheim et al. (2012) observed regrowth of *S. aureus* treated with gemcitabine, even at concentrations significantly above the determined minimum inhibitory concentration values. Mutational frequencies were found to be similar to those found for classical antibiotics. Most of the resistant mutants had mutations in the gene encoding deoxyadenosine kinase that is thought to activate gemcitabine (Jordheim et al., 2012). It was concluded that mutations of deoxyadenosine kinase were important for conferring resistance to gemcitabine, although as some resistant mutants had no mutations in the dNK genes, there are likely to be other mechanisms involved. The induced resistance was found to be highly stable (Jordheim et al., 2012).

While resistance to both zidovudine and gemcitabine arises readily, this could be prevented at least in part either by coadministering with other drugs or possibly by modifying the drug itself. Zidovudine has been shown to have synergistic activity with gentamicin and amikacin (Doleans-Jordheim et al., 2011). Gemcitabine has synergy with gentamicin, and combinations of drugs may help slow the emergence of resistance (Jordheim et al., 2012).

## Conclusions and Future Perspectives

Nucleoside analogues are widely used as effective therapeutics for a variety of diseases and thus make an interesting focus on repurposing studies. Studies to date have yielded promising data on the efficacy of analogues as antibacterial agents, highlighting their potential for use in treating bacterial infections. This potential will become increasingly important as the numbers of multidrug-resistant bacteria continue to rise. Nonetheless, to the best of our knowledge, no nucleoside analogues are currently approved for treating bacterial infections, and so they are not used even in cases where no other options are available.

What are the gaps in current knowledge that are barriers to the use of nucleoside analogues in treating bacterial infections? First and foremost, our review highlights the paucity of studies investigating the effectiveness of nucleoside analogues in treating infections in animal models, information that is needed to determine doses that would be needed to treat infections. This information is particularly crucial for nucleoside analogues that commonly have adverse side effects when used at higher doses, such as in cancer therapy. Second, although the nucleoside analogues discussed here are approved for use in people, clinical trials will be necessary to test their effectiveness in treating bacterial infections and costs are associated with these. Third, the antibacterial modes of action of many nucleoside analogues are not fully understood. Overcoming this knowledge gap may facilitate strategies for better targeting nucleoside analogues as inhibitors of infection while minimizing toxicity to patients. Lastly, although investigations of resistance to nucleoside analogues have been carried out in a few cases, a comprehensive picture is lacking and it is not known how readily resistance might arise during infection. Examples described here of the use of nucleoside analogues in combination with other antibiotics, or as inhibitors of virulence rather than of bacterial growth, suggest strategies that may minimize the development of resistance.

The increasing emergence of multidrug-resistant bacteria capable of causing severe, life-threatening infections provides a hugely powerful imperative to develop new approaches for treating bacterial infections. The research reviewed here highlights the potential for use of nucleoside analogues in situations where classical antibiotics fail. With the current requirement for more antibiotics, it will be very important to fill the knowledge gaps described above, so that nucleoside antibiotics can provide a clinically usable tool in the treatment of bacterial infections.

## Author Contributions

JT and IL reviewed the literature. JT prepared the figures. JT and IL wrote the manuscript.

### Conflict of Interest Statement

The authors declare that the research was conducted in the absence of any commercial or financial relationships that could be construed as a potential conflict of interest.

## Abbreviations

dNK: Deoxyribonucleoside kinase
HPRT: Hypoxanthine-guanine phosphoribosyl transferase
TK: Thymidine kinase.
